# Cardiovascular magnetic resonance stress perfusion imaging in patients with atrial fibrillation

**DOI:** 10.1186/1532-429X-15-S1-E59

**Published:** 2013-01-30

**Authors:** Thomas Hucko, Christoph Klein, Bernhard Schnackenburg, Christopher Schneeweis, Sebastian Kelle, Alexander Berger, Eckart Fleck, Rolf Gebker

**Affiliations:** 1 Internal Medicine - Cardiology, German Heart Institute Berlin, Berlin, Germany; 2 Philips Health Care, Hamburg, Germany

## Background

Several studies have demonstrated the consistently high diagnostic and prognostic value of adenosine stress perfusion imaging with cardiovascular magnetic resonance (CMR). The feasibility and diagnostic accuracy of CMR stress perfusion in patients with atrial fibrillation is unknown. The purpose of this study was to assess the utility of CMR stress perfusion imaging in patients with atrial fibrillation who had suspected and known coronary artery disease (CAD).

## Methods

Thirty-eight patients with suspected myocardial ischemia underwent adenosine stress and rest perfusion CMR (bSSFP, TE/TR/alpha 1.3 ms/ 2.5 ms/ 50°, 3 slices/heartbeat, voxel size 2.9 x 3.0 x 10 mm) and late gadolinium enhancement (LGE) imaging (free breathing single shot, bSSFP, TE/TR/alpha 1.6 ms/ 3.1 ms/ 50°, voxel size 1.8 x 1.9 x 10 mm) at 1.5T. All scans were examined by two independent readers. Image quality was assessed on a 4-point scale (1=excellent, 2=good, 3=moderate, 4=non-diagnostic). Segmental perfusion and LGE images were evaluated for stress induced myocardial ischemia. Perfusion deficits were compared with the results of invasive coronary angiography on both a patient and coronary territory basis. Patients not undergoing invasive coronary angiography were followed up for adverse cardiac events (PCI, CABG, myocardial infarction, cardiac death).

## Results

All patients were examined successfully. Mean image quality of perfusion was 1.73 ± 0.61. Patient- and territory-based inter-observer variability was very low (κ=0.94 and 0.89, respectively). LGE indicating prior myocardial infarction was present in 12 patients with a mean image quality of 2.16 ± 0.69. All patients (n=9) with stress inducible perfusion deficits underwent invasive coronary angiography. A coronary stenosis ≥50% was found in 8 (89%) patients. Of these, 7 (78%) had a stenosis ≥70%. Seven patients underwent invasive angiography despite negative CMR perfusion. Of these, 2 had stenosis ≥50% and none had stenosis ≥70%. The remaining 22 perfusion negative patients were followed up for a mean of 14 ± 7 months (median: 12.9 months). None of these patients experienced a major cardiac event.

## Conclusions

In patients with atrial fibrillation, CMR stress perfusion can detect CAD with good image quality and low inter-observer variability. CMR perfusion imaging may offer an alternative method of detecting ischemia for the purpose of risk stratification and guiding revascularisation in patients with atrial fibrillation.

## Funding

None.

**Figure 1:**
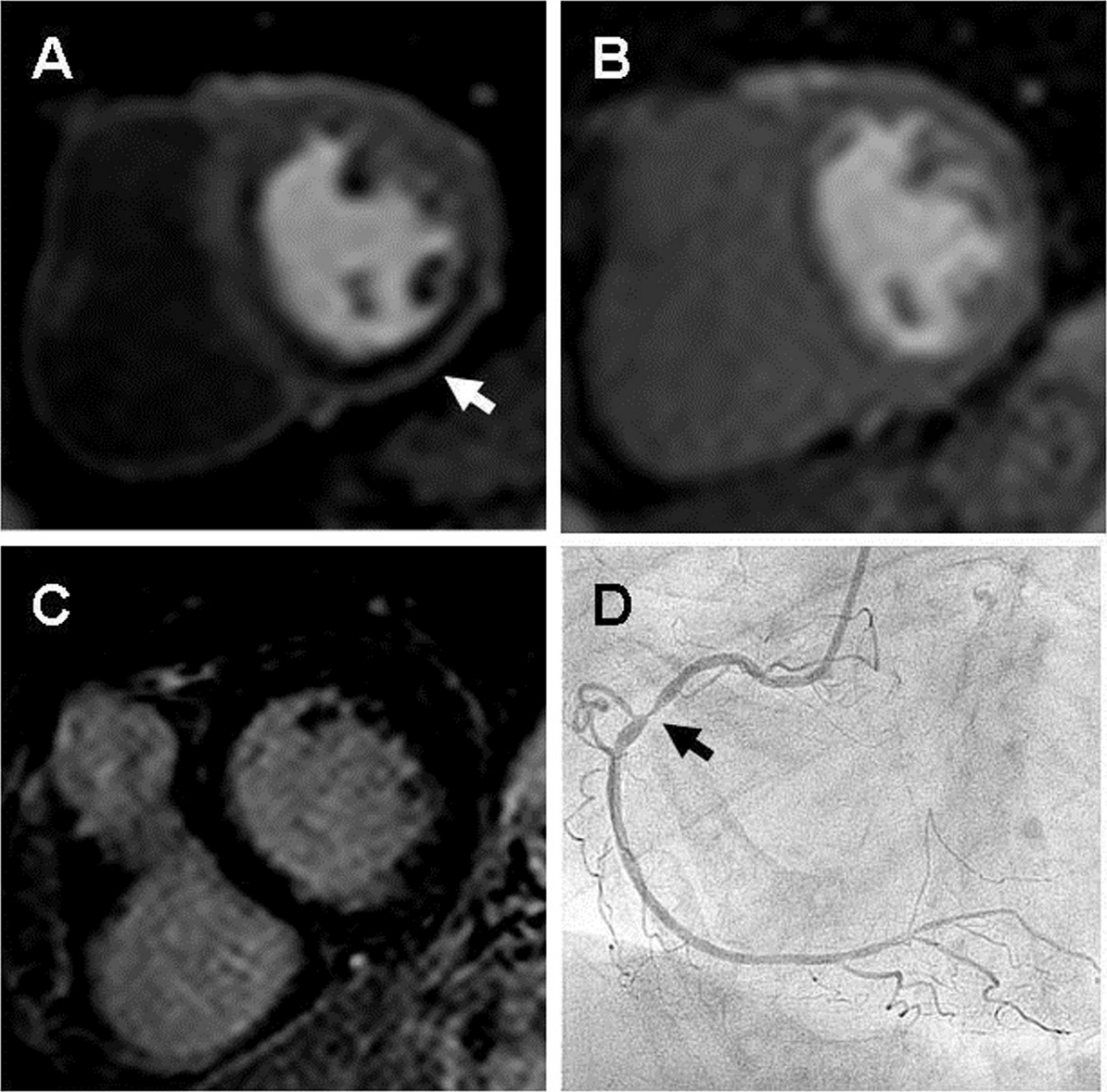
A: basal short axis view with perfusion deficit at stress (arrow), B: basal short axis view without perfusion deficit at rest, C: basal short axis view without late gadolinium enhancement, D: invasive coronary angiography with significant stenosis of proximal RCA (arrow)

